# Self discipline and obesity in Bangkok school children

**DOI:** 10.1186/1471-2458-11-158

**Published:** 2011-03-10

**Authors:** Chutima Sirikulchayanonta, Wasoontara Ratanopas, Paradee Temcharoen, Suwat Srisorrachatr

**Affiliations:** 1 Department of Nutrition, Faculty of Public Health, Mahidol University. 420/1 Rajvithi Road, Rajthevi district, Bangkok 10400, Thailand

## Abstract

**Background:**

Childhood obesity has become an important public health problem in Thailand. This study aimed to determine the relationship between self discipline and obesity in Bangkok school children.

**Methods:**

A case control study was conducted. 140 cases (obese children) and 140 controls (normal weight children) were randomly chosen from grades 4-6 students in 4 Bangkok public schools. Questionnaire responses regarding general characteristics and child self-discipline were obtained from children and their parents.

**Results:**

Self discipline in eating habits, money management and time management were reported at significantly lower levels among the obese group (p < 0.05). After controlling all other variables, it was revealed that the ranking of factors associated with obesity by adjusted odds ratio (OR) were low self-discipline in managing expenses (3.1), poor home environment (3.0,), moderate self-discipline in time management (2.9), television viewing time ≥2 hours/day (2.6), an obese father (2.2), and an obese mother (1.9).

**Conclusions:**

It was recommended that parents and teachers participate in child self-discipline guidance, particularly with regard to eating habits, money management and time management in a supportive environment that both facilitates prevention of obesity and simultaneously develops a child's personal control.

## Background

Childhood obesity is a global epidemic [1]. The prevalence of obese children aged 6-11 years has more than doubled since the 1960s. Results from the 1999-2002 National Health and Nutrition Examination Survey (NHANES), indicated that 15.3 percent of children aged 6-11 years were overweight [2]. In Thailand, a nutritional survey was conducted in Bangkok primary schools between 1992 and 1994 involving 2,885 student respondents. The results showed that obesity prevalence rates had increased from 25.9% to 31.5% in demonstration schools, 25.7% to 28.1% in private schools, 23.3% to 27.4% in government schools, and 11.2% to 14.6% in Bangkok Metropolitan schools[3]. In summary, childhood obesity had become an important public health problem in Thailand, especially in big cities such as Bangkok.

Other studies [4,5] showed that childhood obesity also led to the risk of obesity in adulthood. Long-term health consequences of obesity include type 2 diabetes, cardiovascular disease, hypertension, hyperlipidemia, certain forms of cancer, as well as respiratory and skin problems [6,7]. Obesity in school children was influenced by society, economic conditions, environmental changes, the family's eating habits and child rearing practices[8], leading to unhealthy eating behavior [7] and a sedentary life style characterized by increased television viewing and a lack of physical exercise [9]. It has been reported that poor self-control and low self-discipline are the most important for eating in response to external food stimuli [10,11] leading to obesity.

"Self-discipline", is the ability of an individual to adhere to actions, thoughts, and behavior that result in personal improvement instead of instant gratification[12]. Our research question was, "Are there any differences in self-discipline among obese children compared to children of normal weight?" This research aimed to determine the relationship between self discipline with regard to eating habits, money management, time management and child obesity. It also aimed to analyze the relationship between child obesity and other related family factors (e.g., socioeconomic status, parental weight status, parental guidance in child self-discipline, home environment, television viewing time).

## Methods

A case-control study was conducted in four public schools in Bangkok under the Office of the Basic Education Commission (OBEC), Ministry of Education (MOE), which voluntarily participated in the Bright and Healthy Thai Kid (BAHT) Project from May 2004 to January 2005. These schools were randomly selected from 38 schools of OBEC based on OBEC's zoning system. Two schools were selected from the inner zone, and one each from the middle and outer zones. All four schools were co-educational, and shared similar demographics with regard to gender, numbers of students (1,203, 1,008, 1,591 and 1,324 respectively), family socioeconomic status (low to middle class), parental support, and school environment. All respondents were subject to anthropometric measuring. Weight was measured in kilograms including one decimal point using an electronically calibrated scale (Seca, German) and height was measured in centimeters including one decimal point using a calibrated stadiometer (Microtoise) following a standard measurement [13]. Child weight status was assessed by criteria listed in the INMU Thai Growth program as weight for height (WFH) [14]. The standard criteria were that WFH from -2 SD to 2 SD = normal, WFH greater than 2 SD = obese, and WFH less than -2SD = underweight. This classified the children into 3 weight groups, obese, normal and underweight. A total of 2,585 students who were studying in grades 4-6 were included in the study. The sample size was determined by Schlesselman's formula [15].

A total minimum sample of 135 cases was initially calculated and an additional 5 cases were later added.

### Sample selection

Inclusion criteria

1. Students were studying in grades 4-6 aged 8 -12 years.

2. They were in the academic year 2004 in 4 selected primary schools.

3. They joined the study voluntarily.

4. They fit the obese and normal weight criteria of WFH.

Exclusion criteria

1. Students who were studying in grades 3 or below.

2. Students who were underweight.

**Figure 1:**
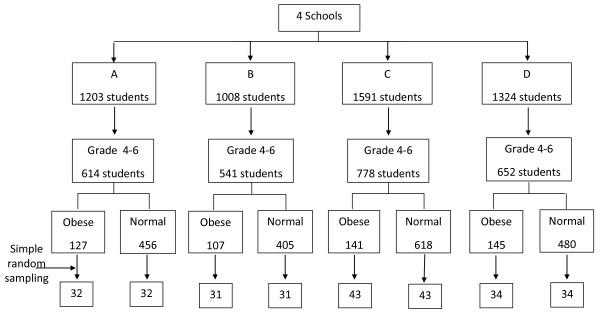
**Sample selection for case and control**.

Simple random sampling was used. First, 140 obese students from grades 4-6 at each school (based on student proportions at the 4 schools) were selected and labeled as a case. Then an equal number of randomly selected children of normal weight from the same classroom (similar demographics with regard to gender, family and socioeconomic status) were drawn and labeled as a control group (Figure 1). Informed consent was obtained from 100% of both the participants and their parents. All protocols for the study were reviewed and approved by the Institutional Review Board, of the Faculty of Public Health, Mahidol University, Proof No. 59/2004.

### Data collection

Two sets of different self-administered questionnaires were used for the collection of data regarding self discipline and obesity. The first set was for the children and the second for their parents. These were in Thai language but have been translated into English in supplementary data (Additional file 1 and 2). The data collected was restricted to the two week period immediately preceding the filling of the questionnaire.

The validity of the questionnaire content was reviewed by experts (staff of the Faculty of Public Health with experience in related fields) with respect to appropriateness for the theme of the research and for the respondents. Then the questionnaires were pre-tested with 30 parents and students at the same schools. Reliability testing using Cronbach's alpha co-efficient method resulted in a reliability level of 0.70.

**Figure 2:**
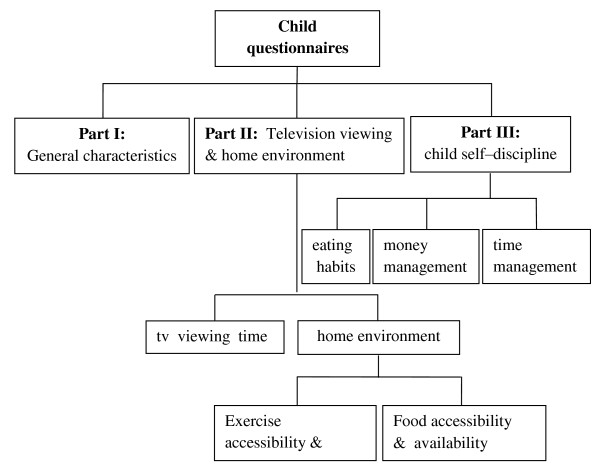
**Child questionnaires**.

1. **Child questionnaires **consisted of 3 parts ( Figure 2) as follows:

**Part I**: General characteristics regarding gender, birth date and number of family members.

**Part II**: Television viewing and home environment was divided into 2 sections as follows:

**Section I**: Television viewing time (hours/day) (1 item)

**Section II**: The home environment questions consisted of 10 items including pertinent questions about accessibility to places of exercise and the availability of exercise equipment (3 items). There were also questions regarding accessibility and availability of various foods both at home and near the home (7 items). Scores of 1 (good environment) or 0 (poor environment) were assigned. From the highest total environmental score of 10 points, results were graded as good (highly supportive of health) at 8-10 points, fair at 6-7.9 points and poor at less than 6 points.

**Part III**: Questions regarding child self-discipline in 3 related areas (24 item in all) were as follows: Area 1 covered eating habits (8 items) including numbers of meals and snacks etc; Area 2 covered money management (6 items) including consideration before purchase, food choices and frugality; Area 3 covered time management (10 items) including a timetable for daily routines with such parameters as "on time" and "set times" for physical exercise and television viewing. Scores for positive health effects were 1 for never/rarely, 2 for sometimes (1-3 days/week), 3 for often (4-6 days/week) and 4 for always (7 days/week). Scores for negative child health effects (e.g., unhealthy eating habits) were scored in the reverse direction. Mean scores in each group were analyzed and were graded into 3 groups for self-discipline as high (3.01-4.00), moderate (2.01-3.00) and poor (1.00-2.00).

The questionnaires were answered by children at schools and if there were any questions they could ask a researcher.

**Figure 3:**
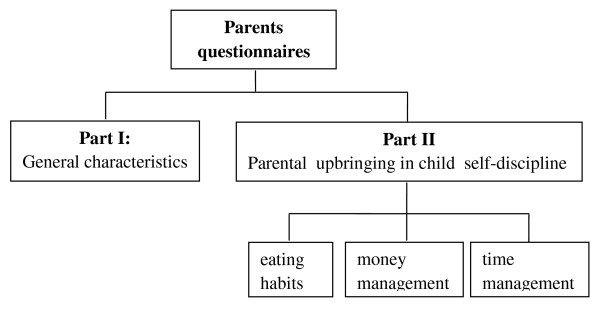
**Parents questionnaires**.

2. **Parent questionnaires **consisted of 2 parts (Figure 3) as follows:

**Part I**: General questions regarding parent characteristics such as weight, height and socioeconomic status (10 items).

**Part II**: Questionnaire on parental guidance in child self-discipline covered 3 areas (24 items) as follows: Area 1 covered child eating habits (12 items), including preparation and provision of healthy meals and snacks. Area 2 covered money management (6 items), including consideration before purchase, food choices and frugality. Area 3 covered time management (6 items) including a timetable for daily routines with such parameters as "on time" and "set times" for physical exercise and television viewing. Scoring and grading was the same as used for Part III of the child questionnaires.

### Statistical analysis

SPSS for Windows, version 11.0 was used. Descriptive statistics were used to describe the general characteristics of children and their parents as numbers, percentages, means, and standard deviations. A Chi-square test was used to examine the relationship between independent variables and childhood obesity in bivariate analysis. A difference was considered statistically significant if the p-value was < 0.05. Multiple logistic regression was utilized to determine the association of independent variables and child obesity adjusting for potential confounding factors including gender, age, parental nutritional status, home environment, child eating habits, money management, time management and television viewing time. Adjusted odds ratios and 95% confidence intervals (CI) were reported.

## Results

There were 140 students in the obese group and another 140 in the normal weight group. The proportions of age and gender (more males than females) were quite similar in both groups (Table 1). In addition, both groups had similar socioeconomic backgrounds (data not shown). There was a higher prevalence of parental obesity reported among parents of the obese group than among the parents of the normal weight group (Table 2). There was a significant relationship in weight status between father, mother and their children (OR = 2.2, 2.3; 95% CI = 1.1-3.3, 1.2-3.3). In other words, the risk of being obese was 2.2 and 2.3 times higher among children who had obese fathers and mothers. Regarding environmental factors, it was shown that the risk of becoming obese was 2.8 times higher among children who had a poorer home environment (OR = 2.8, 95% CI = 1.3-6.1). Furthermore, the risk of becoming obese was 2.7 times higher among children who watched television more than 2 hours per day.

**Table 1:** Number and percentage of children classified by gender and age

	Weight status			
	
Variables	Obese group (n = 140)		Normal weight group (n = 140)	
	
	Number	Percentage	Number	Percentage
**Gender**				
Male	76	54.3	71	50.7
Female	64	45.7	69	49.3
**Age**				
8 - 9 years	83	59.3	86	61.4
10-12 years	57	40.7	54	38.6

**Table 2:** Characteristics of obese and normal weight children

	child's weight status				
				
Variables	Obese(n = 140)	Normal wt (n = 140)	p-value (χ^2^-test)	OR	95%CI for OR
				
	Percentage	Percentage			
**Parental nutritional status**					
**Father**					
Normal wt	22.9	40.0	<0.05	1.0	
Obese	77.1	60.0		2.2	1.1-3.3
**Mother**					
Normal wt	45.7	65.7	<0.05	1.0	
Obese	54.3	34.3		2.0	1.2-3.3
**Home environment**					
Good	8.6	18.6	0.03	1.0	
Moderate	42.9	44.3		2.1	1.0-4.5
Poor	48.6	37.1		2.8	1.3-6.1
**Television viewing time**					
<2 hours/day	25.7	48.6	<0.05	1.0	
≥2 hours/day	74.3	51.4		2.7	1.7-4.5
**Eating habits**			<0.05		
High	34.3	50.7		1.0	
Moderate	60.7	46.4		1.9	1.2-3.12
Low	5.0	2.9		2.5	0.7-9.3
**Managing expenses**			<0.05		
High	17.1	27.1		1.0	
Moderate	65.0	64.3		1.6	0.9-2.9
Low	17.9	8.6		3.3	1.4-7.8
**Time management**			<0.05		
High	8.6	42.9		1.0	
Moderate	65.0	47.1		3.8	2.2-6.6
Low	17.9	10.0		1.8	0.8-4.7

Regarding child self-discipline, the risk of being obese was 1.9 times higher among children who had poor eating habits (OR = 1.9, 95% CI = 1.2-3.1). These included such things as eating more than 3 meals a day, frequently having high caloric food intake such as fried pork, fried eggs, French fries, cakes, snacks and soft drinks. In addition, the risk of becoming obese was 3.3 and 3.8 times higher among children who were poor at managing expenses and time (OR = 3.3 and 3.8; 95% CI = 1.4-7.8 and 2.2-6.6). After controlling all other variables using multiple logistic regression analysis, it was revealed (Table 3) that the first ranking adjusted odds ratio (AOR) for factors associated with childhood obesity was low level self-discipline in managing expenses (AOR 3.1, 95% CI = 1.1-8.2), followed by a poor home environment (AOR 3.0, 95% CI = 1.2-7.5), poor time management (AOR 2.9, 95% CI = 1.6-5.4), long television viewing time (AOR 2.6, 95% CI = 1.5-4.6), and father's and mother's obesity (AOR 2.2 and 1.9; 95% CI = 1.3-3.7 and 1.1-3.4), respectively (p value < 0.05).

**Table 3:** Adjusted Odds ratio (OR) for factors associated with childhood obesity

Variables	Unadjusted OR	95%CI for unadjusted OR	Adjusted OR^#^	95%CI for adjusted OR
Low self-discipline in managing expenses	3.3	1.4-7.8	3.1*	1.1-8.2
Poor home environment	2.8	1.3-6.1	3.0*	1.2-7.5
Moderate self-discipline in time management	3.8	2.2-6.6	2.9*	1.6-5.4
Television viewing time	2.7	1.7-4.5	2.6*	1.5-4.6
≥2 hours/day				
Obese father	2.2	1.1-3.3	2.2*	1.3-3.7
Obese mother	2.0	1.2-3.3	1.9*	1.1-3.4

# After controlling for other variables including.

* Significant at p-value < 0.05

With regard to parental guidance in child self-discipline, it was found that there was no significant difference between the obese and the normal weight groups (data not shown).

## Discussion

Our research findings showed that there were relationships between a child's weight status and low self discipline as regards money management, poor time management, a poor home environment and long television viewing time. A previous study reported that poor self-regulation in early childhood may predispose children to excessive weight gain through early adolescence[16]. This was supported by another study that comprehensive efforts to prevent youth risk for obesity should include target self-regulation and decision-making skills[17].

It is generally accepted that parents play an important role in childhood obesity due to their child rearing practices[18]. Various studies state that obese parents were more likely to have obese children compared to parents of normal weight [19]^,^[20]. This might be explained by genetic transference, energy-dense dietary intake and low levels of physical activity for obese parents [21]. Home environmental factors such as availability and accessibility of food in the home or nearby food shops may also affect the food consumption behavior of children [8] and be positively related to their food choices. Our study also indicated that obese children showed poorer money management when compared to the normal weight group. They most frequently purchased food items that were energy-dense, including low-nutritive foods and beverages such as crispy snacks, fried chicken and sugar-sweetened beverages as reported in another study[22]. From our findings, this factor was associated with more risk of obesity than was parental obesity.

Regarding time management, the obese group spent 2.7 times longer watching television per day than did normal weight children. In other words, they did not manage their time well enough to allow for physical activity. In the home, television is an important source of news, entertainment and advertising, particularly for food. It can attract school children to spend more time watching television and less time exercising. In addition to a decrease in physical activity, there may be a simultaneous increase in energy intake due to the consumption of snacks while watching television. Such behavior causes obesity. Francis [23] reported for families where one or both parents were overweight that children who watched more television also snacked more frequently and consequently had higher intakes of fat from energy-dense snacks. In other words, excessive television viewing and snacking patterns are risk factors for the development of obesity[23]and hyperlipidemia[7] in children. To counter this, Timperio et al [24] reported that sibling engagement in physical activity within the home might be an important target for the prevention of weight gain during the transition from childhood to adolescence. Finnerty et al [25] also investigated the effects of peer influence on physical activity in school children and found that peers have a significant effect on physical activity levels. Familial support including encouragement to eat healthy foods like fruit and vegetables and to do physical exercise would help prevent obesity. Lindelof [26] did a qualitative study involving field observations and semi-structured interviews with 15 obese adolescents and their parents. The results showed that obese adolescents were aware of having unhealthy eating habits and that they wished they were able to attain to a healthier diet. They blamed not only themselves for being obese but also their parents for providing an unhealthy diet or little support for exercise. On the other hand, parents blamed their obese children of lacking will power to change eating and exercise habits. Therefore an obese individual's views including those about a healthier home diet must go together with a positive approach from parents if a lasting change in behavior is to be achieved.

Sirikulchayanonta, et al [27] conducted research on obesity prevention and control by promoting healthy diets and physical exercise in 4 primary schools in Bangkok. It was learned that Thai children were overindulged because of small family sizes. Parents also had positive feelings that overweight children were healthy and cute and that there was social value in eating out. In addition, they believed that teachers were respected by their children and that they would be better able to instruct children about these issues. On the other hand, the teachers believed that child eating behavior originated at home and that health issues were best handled by physicians or paramedics rather than schools. Participatory action research involving students, teachers and parents was carried out to determine whether these basic attitudes could be changed. After a 2-day training course, teachers integrated project concepts into their course curriculum. Seminars on weight management were given separately to parents and students. After 8 months, results showed that high caloric dietary intake significantly decreased, aerobic exercise activity increased (P < .001) and prevalence of obesity declined from 19.3% to 16.8%. Thus, a long-term, participatory effort to promote healthy diets and physical exercise could be effective with primary school students and may establish habits that last to later life.

Regarding sociological theory, a sound explanation why obese children lack self-discipline, watch much television and have poor ability to control their money and time was reviewed. Based on Bourdieu's theory of practice [28], habitus is the product of inculcation and appropriation necessary in order for the products of collective history, to succeed in reproducing themselves more or less complete, in the form of durable dispositions in the individuals lastingly of the same conditionings, and hence placed in the same material conditions of existence. Cited from Lindelof [26] Bourdieu develops habitus to explain the reason for behavioral similarities within different social classes. He proposed that behavior is mediated by habitus, which at a pre-conscious level organizes the individual's behavior in certain patterns reflecting the habitus. Habitus is formed in the individual's past by material, cultural and social conditions, and experiences. However, childhood and youth are of central importance to the formation of habitus. Thus, habitus cannot be grasped as it constantly changes with time and newly integrated experiences. In other words, habitus takes the past and directs it into the future as a specific way of acting in daily life. Therefore sociology treats as identical all the biological individuals who, being the product of the same objective conditions, support the same habitus. Attention must be paid to the underlying mechanism that generates unhealthy behaviour leading to obesity. To achieve a healthier lifestyle for obese individuals, a habitus is needed to stimulate and generate healthier habits.

Limitations of the present study include the self-reported parental weight status and height, that might introduce recall bias and affect the analysis. Some of the questionnaires on self discipline were subjective and some children might not have responded with their actual practices. Thus self-discipline is complex and highly individual and a qualitative exploration involving fieldwork and interviews should be explored in order to get the actual practices and to understand their socio-cultural context and habitus.

## Conclusions

It is recommended that both parents and teachers participate in guidance of school children with regard to self- discipline in eating habits, money management and time management. A supportive environment conducive to healthy eating and physical activity both in the school and at home should be encouraged. A holistic approach should provide primary preventative measures against childhood obesity and future non-communicable diseases. Longitudinal studies for deeper investigations and qualitative approaches to a discussion of social, economic and cultural matters were suggested.

## List of abbreviations

OBEC: Office of the Basic Education Commission; MOE: Ministry of Education; WFH: weight for height; SD: Standard deviation; OR: Odds ratios; AOR: Adjusted Odds ratios; INMU: Institute of Nutrition, Mahidol University; 95% CI: 95% Confidence Interval.

## Competing interests

The authors declare that they have no competing interests.

## Authors' contributions

CS was the principal investigator and made substantial contributions to the conception of the study. CS, PT, WS and SS have made contributions to the design of the study and to the analysis and interpretation of the data. WS was involved in data collection and analysis and drafting of the results. CS revised the content, reviewed and wrote the manuscript and approved the final version. All authors read and approved the final manuscript.

## Pre-publication history

The pre-publication history for this paper can be accessed here:

http://www.biomedcentral.com/1471-2458/11/158/prepub

## Supplementary Material

Additional file 1 **Child Questionnaire**. As requested by the editor, the child questionnaire used in the study was translated into English and presented for the readers. Child questionnaire was consisted of 3 parts: General characteristics; Television viewing and home environment; Self-discipline questionnaire regarding child eating habits, managing expenses, time management. Click here for file

Additional file 2 **Parent questionnaire**. Parent questionnaire was consisted of 2 parts: General characteristics; How parents upbringing their children for self- discipline. The questionnaires were designed by the Bright and Healthy Thai Kid Project Group. Any use of it should be noticed to the Group and must be properly cited in any related research products. Click here for file

## References

[B1] WHO Global Strategy on Diet, Physical Activity and Health 2008 http://www.who.int/features/factfiles/obesity/en/

[B2] Ogden CL Flegal KM Carroll MD Johnson CL Prevalence and trends in overweight among US children and adolescents, 1999-2000 JAMA 2002 288 1728 1732 10.1001/jama.288.14.1728 12365956

[B3] Division Nutrition, Department of Health Situation of Childhood obesity in Thailand 1998 2004 http://advisor.anamai.moph.go.th/factsheet/nutri3-5.htm

[B4] Guo SS Wu W Chumlea WC Roche AF Predicting overweight and obesity in adulthood from body mass index values in childhood and adolescence Am J Clin Nutr 2002 76 653 658 12198014 10.1093/ajcn/76.3.653

[B5] Whitaker RC Wright JA Pepe MS Seidel KD Dietz WH Predicting obesity in young adulthood from childhood and parental obesity N Engl J Med 1997 337 869 873 10.1056/NEJM199709253371301 9302300

[B6] World Health Organization Global Strategy on Diet, Physical Activity and Health. Childhood overweight and obesity 2009 http://www.who.int/dietphysicalactivity/childhood/en/

[B7] Sirikulchayanonta C Pavadhgul P Chongsuwat R Srisorrachata S A preliminary study of hyperlipidemia in Bangkok school children Asia Pac J Public Health 2006 18 15 19 10.1177/10105395060180030401 17153077

[B8] Maffeis C Aetiology of overweight and obesity in children and adolescents Eur J Pediatr 2000 159 Suppl 1 S35 44 10.1007/PL00014361 11011954

[B9] Ma GS Li YP Hu XQ Ma WJ Wu J Effect of television viewing on pediatric obesity Biomed Environ Sci 2002 15 291 297 12642985

[B10] Tiggemann M Rothblum ED Gender differences in social consequences of perceived overweight in the United States and Australia Sex Roles 1988 18 75 86 10.1007/BF00288018

[B11] Elfhag K Morey LC Personality traits and eating behavior in the obese: poor self-control in emotional and external eating but personality assets in restrained eating Eat Behav 2008 9 285 293 10.1016/j.eatbeh.2007.10.003 18549987

[B12] Bukkapatnam M Study Guides and Strategies: Developing self-discipline 2005 http://www.studygs.net/discipline.htm

[B13] Gibson RS Gibson RS Anthropometric assessment of body size Principles of nutritional assessment 2005 2 New York: Oxford University Press 247 253

[B14] Institute of Nutrition, University Mahidol INMU Thai Growth program for nutritional assessment (using weight for height references from national survey 2002 Department of Health, Ministry of Public Health)

[B15] Schlesselman JJ Schlesselman JJ, Stolley PD Sample size Case - Control studies: Design, Conduct, Analysis 1982 New York: Oxford University Press 144 145

[B16] Francis LA Susman EJ Self-regulation and rapid weight gain in children from age 3 to 12 years Arch Pediatr Adolesc Med 2009 163 297 302 10.1001/archpediatrics.2008.579 19349557 PMC9433163

[B17] Riggs NR Sakuma KL Pentz MA Preventing risk for obesity by promoting self-regulation and decision-making skills: pilot results from the PATHWAYS to health program (PATHWAYS) Eval Rev 2007 31 287 310 10.1177/0193841X06297243 17478630

[B18] Hennessy E Hughes SO Goldberg JP Hyatt RR Economos CD Parent behavior and child weight status among a diverse group of underserved rural families Appetite 2010 54 369 377 10.1016/j.appet.2010.01.004 20079785

[B19] Fuiano N Rapa A Monzani A Pietrobelli A Diddi G Limosani A Bona G Prevalence and risk factors for overweight and obesity in a population of Italian schoolchildren: a longitudinal study J Endocrinol Invest 2008 31 979 984 19169053 10.1007/BF03345635

[B20] Lee K Kwon ER Park TJ Park MS Lenders CM Parental overweight as an indicator of childhood overweight: how sensitive? Asia Pac J Clin Nutr 2006 15 196 200 16672203

[B21] Davison KK Birch LL Obesigenic families: parents' physical activity and dietary intake patterns predict girls' risk of overweight Int J Obes Relat Metab Disord 2002 26 1186 1193 10.1038/sj.ijo.0802071 12187395 PMC2530921

[B22] Borradaile KE Sherman S Vander Veur SS McCoy T Sandoval B Nachmani J Karpyn A Foster GD Snacking in children: the role of urban corner stores Pediatrics 2009 124 1293 1298 10.1542/peds.2009-0964 19822591

[B23] Francis LA Lee Y Birch LL Parental weight status and girls' television viewing, snacking, and body mass indexes Obes Res 2003 11 143 151 10.1038/oby.2003.23 12529497 PMC2530922

[B24] Timperio A Salmon J Ball K Baur LA Telford A Jackson M Salmon L Crawford D Family physical activity and sedentary environments and weight change in children Int J Pediatr Obes 2008 3 160 167 10.1080/17477160801970385 19086186

[B25] Finnerty T Reeves S Dabinett J Jeanes YM Vogele C Effects of peer influence on dietary intake and physical activity in schoolchildren Public Health Nutr 2010 13 376 383 10.1017/S1368980009991315 19719887

[B26] Lindelof A Neilsen CV Pedersen BD Obesity treatment more than food and exercise: a qualitative study exploring obese adolescents' and their parents' views on the former's obesity Int J Qualitative Stud Health Well-being 2010 5 5073 10.3402/qhw.v5i2.5073 PMC2875969 20640019

[B27] Sirikulchayanonta C Pavadhgul P Chongsuwat R Klaewkla J Participatory action project in reducing childhood obesity in Thai primary school Asia Pac J Public Health OnlineFirst 2010 10.1177/1010539510361965 20460295

[B28] Bourdieu P Outline of a theory of practice 1993 Cambridge: Cambridge University Press

