# Supplementary material for: Self discipline and obesity in Bangkok school children
Source: BMC Public Health. 2011 Mar 10;11:158. doi: 10.1186/1471-2458-11-158 (PMC3063828; doi:10.1186/1471-2458-11-158)
Supplement: Additional file 1 — Child Questionnaire. As requested by the editor, the child questionnaire used in the study was translated into English and presented for the readers. Child questionnaire was consisted of 3 parts: General characteristics; Television viewing and home environment; Self-discipline questionnaire regarding child eating habits, managing expenses, time management. [file 1471-2458-11-158-S1.DOC]

**Additional file I**

Child Questionnaire

Name ... ... ... ... ... ... ... ………………….class ..... ..............school ........... ... ....

**Part I:** General characteristics
Please check  **/** in parentheses ( ) or fill in the blanks.
1. Sex ( ) Male ( ) female
2. Date / Month / Year ......................................... ..........................
3. Family members ... ... ... ... ... ... ... ... ... ... (including the students).

**Part II:** Television viewing and home environment (exercise, sports facilities offering at home or places around 500 meter away from home ).
 **Section I**: Television viewing time

Actual television viewing time.. ... .. hours .. minutes per day

**Section II:** The home environment. Are there any of the followings?

Please check  **/** in parentheses ( )

| No | Environment | yes | no |
| --- | --- | --- | --- |
| 1. | Playground near your house | ( ) | ( ) |
| 2. | Fitness equipment in your house. | ( ) | ( ) |
| 3. | Playground in your house. | ( ) | ( ) |
| 4. | Local food shops selling fried sausage or fish balls | ( ) | ( ) |
| 5. | Local stores selling candy, crispy snack near your house | ( ) | ( ) |
| 6 | Local stores selling soft drink, ice cream near your house | ( ) | ( ) |
| 7 | Fast food shops such as KFC, Mc Donald Pizza near your house | ( ) | ( ) |
| 8. | Convenience stores like Seven Eleven, etc. near your house | ( ) | ( ) |
| 9. | Local stores selling cake or doughnut near your house | ( ) | ( ) |
| 10. | Local stores selling traditional Thai dessert near your house | ( ) | ( ) |

**Part III:** Self–discipline questionnaire regarding your eating habits, managing expenses, and time management.

| No | List of practice | Always  ( 7 days/  week) | Often  (4-6 days/ week) | Sometimes  (1-3 days/ week) | Never or rarely |
| --- | --- | --- | --- | --- | --- |
|  | **Eating habits** |  |  |  |  |
| 1. | Having breakfast |  |  |  |  |
| 2. | Having lunch |  |  |  |  |
| 3. | Having dinner |  |  |  |  |
| 4. | Having 3 meals with 5 food groups on time |  |  |  |  |
| 5. | Having dessert or snack before staple meal |  |  |  |  |
| 6. | **Having meal or snack before bedtime** |  |  |  |  |
| 7. | **Choosing a meal or snack as advertising** |  |  |  |  |
| 8. | Eating snack while watching TV |  |  |  |  |
|  | Managing expenses |  |  |  |  |
| 9. | Regard to price before buying food or snack. |  |  |  |  |
| 10. | Choosing nutritious food when buying. |  |  |  |  |
| 11. | Spending all the daily pocket money |  |  |  |  |
| 12. | When you have money left over from school, you save it in the piggy bank |  |  |  |  |
| 13. | **You buy food or snacks because of the given small toy premium inside.** |  |  |  |  |
| 14. | You have a plan proportionately to spend money for meals, transportation and snack. |  |  |  |  |

| No | List of practice | Always  ( 7 days/  week) | Often  (4-6 days/ week) | Sometimes  (1-3 days/ week) | Never or rarely |
| --- | --- | --- | --- | --- | --- |
|  | Time management |  |  |  |  |
| 15. | You have your bowel habit nearly the same time each day such as in the morning. |  |  |  |  |
| 16. | **Wake up by yourself.** |  |  |  |  |
| 17. | **Go to bed after watching TV on an evening drama (8.00PM).** |  |  |  |  |
| 18. | Do exercise. |  |  |  |  |
| 19. | **Finish homework before watching television.** |  |  |  |  |
| 20. | **Finish homework before playing games.** |  |  |  |  |
| 21. | Help parents do housework (sweeping, dishwashing, watering plants, etc.). |  |  |  |  |
| 22. | **Self - organized schedule.** |  |  |  |  |
| 23. | **Sleep on average at least a day.** **8-10 hours.** |  |  |  |  |
| 24. | When having free time, you play computer games. |  |  |  |  |
