# Supplementary material for: Self discipline and obesity in Bangkok school children
Source: BMC Public Health. 2011 Mar 10;11:158. doi: 10.1186/1471-2458-11-158 (PMC3063828; doi:10.1186/1471-2458-11-158)
Supplement: Additional file 2 — Parent questionnaire. Parent questionnaire was consisted of 2 parts: General characteristics; How parents upbringing their children for self- discipline. The questionnaires were designed by the Bright and Healthy Thai Kid Project Group. Any use of it should be noticed to the Group and must be properly cited in any related research products. [file 1471-2458-11-158-S2.DOC]

**Additional file ll**

Parent questionnaire

Name ... ... ... ... ... ... ... ……………class ..... ..............school ........... ... ....

Part 1. General characteristics of parents.
Please fill in the blanks and check **/** in parentheses ( ) in front of the actual answer.

1. The weight of his father ... ... ... ... ... .... Kg Height ... ...... ... ... ... .. cm
2. The weight of the mother ... ... ... ... ... ...Kg Height ... ... ... ... ... ... .. cm
3. The highest education of father
     ( ) Less than elementary ( ) Vocational diploma.
     ( ) Elementary ( ) Bachelor's degree.
     ( ) High school ( ) Higher than bachelor

 4. The highest education of the mother.
     ( ) Less than elementary ( ) Vocational diploma.
     ( ) Elementary ( ) Bachelor's degree.
     ( ) High school ( ) Higher than bachelor

5. Father’s occupation

     ( ) Not working ( ) state employee / private companies / banks.
     ( ) Employee ( ) a private business.
     ( ) Government officer ( ) Other specify ... ... ... ... ... ... ... ... ...
6. Mother’s occupation
     ( ) Not working ( ) state employee / private companies / banks.
     ( ) Employee ( ) a private business.
     ( ) Government officer ( ) Other specify ... ... ... ... ... ... ... ... ...

7. The average monthly income of families.
     ( ) <5,000 baht ( ) 10,001 to 20,000 Baht
     ( ) 5001 - 10,000 baht ( ) > 20,000 Baht

Part 2. How parents upbringing their children for self- discipline. (Your practicing **during the last 2 weeks**).

Please check **/** in the space.

| No | List of practicing | Always 7d/wk | Often  4-6d/ wk | Sometimes  1-3d/wk | Never/  rarely |
| --- | --- | --- | --- | --- | --- |
|  | **Dietary practice** |  |  |  |  |
| 1 | You provide 5 food groups for your children. |  |  |  |  |
| 2. | You and your family have 3 meals a day at usual time together. |  |  |  |  |
| 3. | You recommend your children to eat food just proper portion size for them. |  |  |  |  |
| 4. | You encourage your children to eat vegetables. |  |  |  |  |
| 5. | You keep fruit in the refrigerator at home. |  |  |  |  |
| 6. | Taking your children to eat out (buffet). |  |  |  |  |
| 7 | Keeping crispy snacks in the home. |  |  |  |  |
| 8 | Having soft drinks or carbonated beverage in the refrigerator. |  |  |  |  |
| 9 | You take your children to fast food restaurants. |  |  |  |  |
| 10 | Providing fried food for your children. |  |  |  |  |
| 11 | You provide food cooked with coconut milk to your children. |  |  |  |  |
| 12 | You provide soft drinks and snacks to children during television viewing. |  |  |  |  |

| No | List of practicing | Always | Often | Some-times | Never/  rarely |
| --- | --- | --- | --- | --- | --- |
|  | **Money management** |  |  |  |  |
| 13. | Train your children in consideration before buying things (is it necessary to buy?). |  |  |  |  |
| 14. | Teach your children how to buy nutritious food. |  |  |  |  |
| 15. | Teach your children in money-saving. |  |  |  |  |
| 16. | Teach your children to compare prices and quality before buying things. |  |  |  |  |
| 17. | Allow your children buying all kinds of food or snack they like. |  |  |  |  |
| 18 | Bring your children to the shopping mall only if necessary. |  |  |  |  |
|  | **Time management** |  |  |  |  |
| 19 | You train your children in having bowel habit in the morning. |  |  |  |  |
| 20 | You tell your children to go to bed at usual time. |  |  |  |  |
| 21 | Exercise together with your children. |  |  |  |  |
| 22 | Set the rule for your children to finish homework before watching TV. |  |  |  |  |
| 23 | Tell your children to finish homework before playing games. |  |  |  |  |
| 24 | Let your children do some housework. |  |  |  |  |
